# An increase in visceral fat is associated with a decrease in the taste and olfactory capacity

**DOI:** 10.1371/journal.pone.0171204

**Published:** 2017-02-03

**Authors:** Jose Carlos Fernandez-Garcia, Juan Alcaide, Concepcion Santiago-Fernandez, MM. Roca-Rodriguez, Zaida Aguera, Rosa Baños, Cristina Botella, Rafael de la Torre, Jose M. Fernandez-Real, Gema Fruhbeck, Javier Gomez-Ambrosi, Susana Jimenez-Murcia, Jose M. Menchon, Felipe F. Casanueva, Fernando Fernandez-Aranda, Francisco J. Tinahones, Lourdes Garrido-Sanchez

**Affiliations:** 1 CIBER Fisiopatología Obesidad y Nutrición (CIBERObn), Instituto Salud Carlos III, Madrid, Spain; 2 Unidad de Gestión Clínica de Endocrinología y Nutrición, Instituto de Investigación Biomédica de Málaga (IBIMA), Hospital Clínico Virgen de la Victoria, Málaga, Spain; 3 Department of Psychiatry, University Hospital of Bellvitge-IDIBELL, Barcelona, Spain; 4 Department of Psychological, Personality, Evaluation and Treatment of the University of Valencia, Valencia, Spain; 5 Department of Basic Psychology, Clinic and Psychobiology of the University Jaume I, Castelló, Spain; 6 Human Pharmacology and Clinical Neurosciences Research Group, Neuroscience Research Program, IMIM (Hospital del Mar Medical Research Institute), Barcelona, Spain; 7 Department of Experimental and Health Sciences, Universitat Pompeu Fabra Barcelona, Spain; 8 Department of Diabetes, Endocrinology and Nutrition, Institutd’Investigació Biomèdica de Girona (IdlBGi) Hospital Dr Josep Trueta, Girona, Spain; 9 Metabolic Research Laboratory, Clínica Universidad de Navarra, University of Navarra-IdiSNA, Pamplona, Spain; 10 Department of Clinical Sciences, School of Medicine, University of Barcelona, Barcelona, Spain; 11 CIBER Salud Mental (CIBERSAM), Instituto Salud Carlos III, Barcelona, Spain; 12 Endocrine Division, Complejo Hospitalario U. de Santiago, Santiago de Compostela University, Santiago de Compostela, Spain; The University of Tokyo, JAPAN

## Abstract

**Introduction:**

Sensory factors may play an important role in the determination of appetite and food choices. Also, some adipokines may alter or predict the perception and pleasantness of specific odors. We aimed to analyze differences in smell–taste capacity between females with different weights and relate them with fat and fat-free mass, visceral fat, and several adipokines.

**Materials and methods:**

179 females with different weights (from low weight to morbid obesity) were studied. We analyzed the relation between fat, fat-free mass, visceral fat (indirectly estimated by bioelectrical impedance analysis with visceral fat rating (VFR)), leptin, adiponectin and visfatin. The smell and taste assessments were performed through the "Sniffin’ Sticks" and "Taste Strips" respectively.

**Results:**

We found a lower score in the measurement of smell (TDI-score (Threshold, Discrimination and Identification)) in obese subjects. All the olfactory functions measured, such as threshold, discrimination, identification and the TDI-score, correlated negatively with age, body mass index (BMI), leptin, fat mass, fat-free mass and VFR. In a multiple linear regression model, VFR mainly predicted the TDI-score. With regard to the taste function measurements, the normal weight subjects showed a higher score of taste functions. However a tendency to decrease was observed in the groups with greater or lesser BMI. In a multiple linear regression model VFR and age mainly predicted the total taste scores.

**Discussion:**

We show for the first time that a reverse relationship exists between visceral fat and sensory signals, such as smell and taste, across a population with different body weight conditions.

## Introduction

Obesity has become a growing epidemic and is considered the most serious public health issue in developed countries [1]. Recent data show that 1.46 billion people worldwide are overweight and nearly 502 million are obese. Furthermore, in the past 3 decades the prevalence of obesity has doubled and the mean body mass index (BMI) has increased by 0.4 kg/m2 per decade [2].

Obesity has a multifactorial etiology where genetics play a major role, but several environmental shared and non-shared factors also have a significant influence [3]. Besides metabolic and lifestyle factors, a difference in taste and smell perception between obese and normal-weight individuals has been suggested to cause differences in food choices and energy consumption [4]. In obesity, different factors may influence food intake and thereby affect energy balance: sensory signals such as smell, taste and other physical properties of food and internal metabolic signals, such as glucose levels and other hormonal or metabolic changes [5]. Under experimental conditions, the intake of a specific food relative to the intake of other foods is strongly affected by sensory-specific satiety [6]. Sensory systems, and especially smell and taste, plays a significant role in the enjoyment of food and the control of food intake [7]. It has been shown that increased intake and decreased taste or smell in obesity appears to have parallel patterns in both animal [8] and human studies [9]. Other studies show discordant results [10,11]. Bariatric surgery affected sweet taste behavior in rats, with postsurgical rats having lower sensitivity or avidity for sucrose than sham-treated control rats [10]. However, in other study the acuity for sweet taste increases after bariatric surgery leading to increased intensity of perception. [11]. The findings showed that RYGB Different studies have shown a significant correlation between elevated BMI and the presence of olfactory dysfunction in obese subjects [12,13]. In general it has been observed that obese patients have a greater predilection for sweet gustatory stimuli when compared with healthy controls [14,15]. However, studies comparing the taste thresholds and perceived intensities of different weight groups are scarce [16–18].

Further, there are studies which support the concept that peripheral adipokines may alter or predict the perception and pleasantness of specific odors. Recent animal studies have shown that metabolic factors including high blood glucose, insulin or leptin resistance enhance taste response to sweet stimuli administered orally [19,20]. The decrease of serum leptin levels was associated with an improvement of sweet taste sensitivity during weight loss in healthy and obese females [21]. In mice, leptin could modulate olfactory-mediated preingestive behavior through leptin receptors [22]. All these studies appear to support the role of leptin in regulating nutrition, body weight, and energy balance [23]. Improved knowledge on links between odor perception and peripheral metabolism could lead to useful insight for the general application of odors and flavors on metabolism regulation [24].

Thus, our hypothesis is that fat mass may be related to sensory abilities (namely taste and smell) and to different patterns of adipokine secretion. Thus, after considering the limitations of the existing literature, the aim of our study was to analyze the differences in smell and taste between female subjects with different degrees of weight (low-weight, normal-weight, overweight, obese and morbid obese subjects) and relate them to body composition (fat mass, fat-free mass and visceral fat) and adipokine patterns (adiponectin, leptin and visfatin).

## Materials and methods

### Sample

Seven centers, all involved in the Spanish Biomedical Research Network of Obesity and Nutrition (CIBERobn: www.ciberobn.com), participated. Enrolment in the study was between January 2010 and March 2013. All participants gave written and signed informed consent, the study was conducted according to the Declaration of Helsinki, and the Ethics Committee of all the institutions involved [Comissió Deontológica de la Universitat Jaume I; Subcomisión Clínica del Hospital Universitario “Virgen de la Victoria”, Málaga; Comite Etic de Investigacio Clinica Hospital Universitari de Girona Doctor Josep Trueta (048/10); Comite Etico de Investigacion Clinica del Consorci Mar Parc de Salut de Barcelona-Parc de Salut Mar (2010/3914/I); Comité de Etica de la Investigación Universidad de Navarra (110/2010); and Comité Etico de Investigación Clínica del Hospital Universitari de Bellvitge (307/06)] approved the study.

The total sample comprised 179 women, distributed according to BMI (17 low-weight (BMI<18.5 kg/m^2^), 77 normal-weight (BMI = 18.5–24.9 kg/m^2^), 12 overweight (BMI = 25–29.9 kg/m^2^), 28 obese (BMI = 30–39.9 kg/m^2^) and 45 morbid obese (MO)) (BMI≥40 kg/m^2^).

Participants were recruited through several sources including word-of-mouth and advertisements in the local university. The lifetime history of health or mental illness profile was based on the general health questionnaire (GHQ)-28. Prior to assessment, subjects were specifically asked about lifetime or current presence of drug or alcohol abuse or dependence (including cannabis abuse/dependence).

Exclusion criteria were: (1) history of chronic medical illness or neurological condition that might affect cognitive function or olfactory-taste tests (e.g. nasal illness or surgery, hypo/hypergonadism); (2) head trauma, learning disability or mental retardation; (3) being male; (4) having used psychoactive medications or drugs that may interfere with the olfactory-taste capacity and appetite/hunger feeling; (5) having any substance abuse/dependence (including cannabis); (6) age under 18 or over 65 (to discard neuropsychological deficits associated with age); (7) having type I or type II diabetes mellitus (fasting glucose ≥126 mg/dl or medication for type II diabetes).

### Procedures and assessment

The first interview provided information about antecedents and other clinical and health data of interest, as well as the assessment of exclusion and inclusion criteria for the study. In addition to the first clinical interview, basic anthropometrical features and body composition were measured. During the second interview, a comprehensive olfactory and gustatory assessment was made by means of Sniffin' Sticks [25] and Taste Strips [26]. For the olfactory-taste assessment, individuals were requested not to have smoked, chewed gum or eaten any product during the 60 minutes prior to the test. Finally, patients were programmed in a third visit to the hospital for a complete blood analysis. Blood samples from all subjects were collected after a 12-hour fast into blood collection tubes with a serum separator. The serum was separated and immediately frozen at –80°C.

### Olfactory and gustatory assessment

Olfactory testing was performed using "Sniffin' Sticks" (Burghart Messtechnik GmBh, Wedel, Germany). Sniffin' Sticks is a test battery of nasal chemosensory performance that has been previously described and validated [25,27] and it is deemed suitable for the routine clinical assessment of olfactory performance. This test includes subtests for odor threshold, odor discrimination and odor identification. The test consisted of felt-tip pen-like odor dispensers (sticks). Odor threshold was assessed by a triple-forced-choice paradigm. Three pens were presented to the patient in a randomized order, two contained odorless solvent (propylenepropylene glycol) and the other an odorant in a specific dilution (n-butanol). The patient’s task was to indicate the pen with the odorant. Concentration was increased if one of the blanks was chosen and decreased if the correct pen was identified twice in a row. A total of 16 odor concentrations were tested starting from a 4% stock solution (dilution ratio 1:2 solvent propylene glycol). Presentation of the triplets to a subject occurred until they had correctly discerned the odorant in two successive trials which triggered a reversal of the staircase. Threshold is defined as the mean of the last 4 staircase reversal points of a total of seven reversals. Subjects’ scores range between 1 and 16. The higher is the score the higher is olfactory threshold capacity (detection of odor with less solution concentration). The second subtest assessed the ability of the patient to discriminate different odors. Again 16 sets of 3 pens were offered, each including two identical odors and a different one. The patient’s task was to indicate the pen which had a different smell. The score was the sum of correct responses ranging from 0 to 16. The higher is the score the higher is olfactory discrimination capacity. Both threshold and discrimination testing was performed with the patient being blindfolded. For testing odor identification, 16 sticks containing common odors were offered. The patient had to identify each of the odorants from a list of four descriptors. The total score was the sum of correct responses, which can range from 0 to 16 points. The higher is the score the higher is olfactory identification capacity. The odorants used in the discrimination and in the identification Siniffin' Sticks subtests are common odors and similar with regard to both intensity and hedonic tone (orange, peppermint, turpentine, cloves, leather, banana, garlic, rose, fish, lemon, coffee, anise, cinnamon, liquorice, apple, pineapple). The sum of the scores from the three subtests resulted in the composite TDI-score (range from 0 to 48 points.), which is the sum of the results obtained for threshold, discrimination and identification measures [28]. The higher is the score the higher is olfactory capacity. As defined in previous report [29], a TDI score of 30.5 points or more indicates normosmia, a score between 16.5 and 30 points indicates reduced smell function in terms of hyposmia, and a score of less than 16.5 points indicates a smell functional impairment or anosmia. The order for the subtest was always the same. First we started testing threshold, followed by the odor discrimination test and lastly the odor identification test, as the manual of the sniffin' Sticks recommended. The timing between each subtest was approximately 10 minutes.

Gustatory assessment was performed using the “Taste strips”. This reliable and validated test [25] consisted of 16 taste impregnated filter papers of a length of 8 cm with a tip area of 2 cm2. Each of the 16 taste stimuli was impregnated with one of these four tastes: sweet, sour, salty and bitter. The following concentrations were used for the taste stimuli: sweet: 0.4, 0.2, 0.1, 0.05 g/ml sucrose; sour: 0.3, 0.165, 0.09, 0.05 g/ml citric acid; salty: 0.25, 0.1, 0.04, 0.016 g/ml sodium chloride; bitter: 0.006, 0.0024, 0.0009, 0.0004 g/ml quinine hydrochloride. Distilled water was used as the solvent. The strips were placed on the left and right sides of the anterior third of the extended tongue. Before each administration of a strip, the mouth was rinsed with water. The tastes were presented in increasing concentrations. With their tongue still extended, patients had to identify the taste from a list of four descriptors. i. e. sweet, sour, salty and bitter (multiple forced-choice). Each correct answer was granted as 1 point (maximum 4 points for each taste score and 16 points for the whole test score). The number of correctly identified tastes was summed up for a "taste score". Taste Strips has shown usefulness in the clinical practice [25,30] as well as several advantages, such as short time needed for testing, good reproducibility of the results, and the possibility to test each side of the tongue separately. Normative data on Taste strips are based on a sample over 500 participants [30]. Normogeusia was defined as a test score of 9 and higher, while a score below 9 was considered a sign of hypogeusia [26].

### Anthropometrical, biochemical and hormonal measures

Body Composition was assessed using the Tanita Multi-Frequency Body Composition Analyzer MC-180MA (Tanita Corporation, Tokyo, Japan), a weighing instrument which uses bioelectrical impedance analysis for the screening of body fat and composition. This instrument is repeatedly checked in relation to the reference standards of dual-energy X-ray absorptiometry (DEXA) (http://www.bl-biologica.es/tanita_tbf.htm) and has been validated against other weighing methods [31].

Fat mass (Kg) was measured and visceral fat was calculated with the impedaciometer. Visceral fat was indirectly estimated and results were given as a specific rating: visceral fat rating (VFR) (0–59) (no units). Ratings from 1 to 12 indicate that the subject has a healthy level of visceral fat, while ratings from 13 to 59 indicate that the patient has an excess level of visceral fat. Visceral fat rating is been extensively used in medical research as an indirect visceral fat measurement in adults [32] and children [33].

Blood samples from all subjects were collected after a 12-hour fast into blood collection tubes with a serum separator. After 15 minutes, tubes were centrifuged at 4000 r.p.m. at room temperature. The serum was separated and immediately frozen at –80°C. Serum biochemical variables were measured in duplicate, as previously described [34,35]. Adiponectin and leptin levels were measured by means of enzyme immunoassay (ELISA) kits (BioVendor Research and Diagnostic Products, Czech Republic, Europe). Visfatin levels were measured by the ELISA kit from Phoenix Pharmaceuticals (California, USA). Insulin was analyzed by an immunoradiometric assay (BioSource International, Camarillo, CA, USA). The Homeostasis Model Assessment of insulin resistance (HOMA-IR) was calculated from fasting insulin and glucose with the following equation: HOMA-IR = fasting insulin (μIU/mL) x fasting glucose (mmol/L)/22.5.

### Statistical analysis

The statistical analysis was done with SPSS (Version 20 for Windows; SPSS. Chicago. IL). Comparison between the groups was performed with the ANOVA test. Comparison between related variables was performed with the Friedman test (total taste strips, sweet, salty, sour and bitter). The Pearson correlation coefficients were calculated to estimate the correlations between variables. A multivariate analysis was also performed with age, BMI, leptin, fat mass, fat-free mass, VFR and smoking as the independent variables and TDI-score as the dependent variable to determine those factors involved in the abnormality of smell. A similar analysis was performed by replacing the TDI-score with total taste strips as the dependent variable to evaluate taste alterations. Values were considered to be statistically significant when the *P*≤0.05. The results of tables are given as the mean ± SD, and mean ± SEM in figures.

## Results

### Anthropometric, biochemical and hormonal characteristics

Table 1 summarizes the anthropometric, biochemical and hormonal characteristics of the different groups depending on the diagnosis. All participants were female, aged between 18 and 65 years. As expected, significant differences were observed in all the variables based on the diagnosis.

**Table 1:** Anthropometric and biochemical variables in patients included in the study according to the diagnosis.

VARIABLES	LOW WEIGHT (n = 17)	NORMAL WEIGHT (n = 77)	OVERWEIGHT(n = 12)	OBESITY (n = 28)	MO (n = 45)
**Age (years)**	23.1±6.7^c^	27.1±7.3^c^	33.6±8.7^b^	46.4±12.2^a^	42.3±10.7^a^
**Weight (Kg)**	48.5±5.3^e^	59.1±6.6^d^	70.7±5.8^c^	91.6±8.3^b^	120.9±18.3^a^
**BMI (kg/m**^**2**^**)**	17.9±0.51^e^	21.6±1.7^d^	26.8±0.90^c^	35.2±2.6^b^	46.3±5.1^a^
**Glucose (mg/dL)**	85.6±6.8^b^	86.2±18.9^b^	87.5±3.7^b^	101.6±25.6^a^	102.2±19.8^a^
**Cholesterol (mg/dL)**	171.8±35.8^b^	169.3±31.6^b^	190.8±34.8^a.b^	200.5±49.7^a^	192.7±54.4^a.b^
**Triglycerides (mg/dL)**	66.0±23.4^b^	76.9±28.9^b^	88.3±44.6^b^	132.1±82.3^a^	160.2±77.5^a^
**HDL-c (mg/dL)**	67.8±13.8^a^	63.9±12.9^a^	62.3±16.2^a^	53.0±11.9^b^	43.9±12.0^c^
**Insulin (μIU/ml)**	5.7±2.2^c^	7.0±2.9^b,c^	7.8±3.8^b,c^	12.9±7.4^b^	22.6±14.9^a^
**HOMA-IR**	1.2±0.54^c^	1.5±0.67^c^	1.7±0.82^c^	3.3±2.2^b^	5.9±4.8^a^
**Leptin (ng/mL)**	7.0±2.7^e^	15.2±8.5^d^	27.6±15.1^c^	38.3±12.7^b^	44.9±11.3^a^
**Adiponectin (μg/mL)**	11.3±3.9^b.c^	12.7 ±4.4^a,b^	14.4±5.2^a^	10.1±3.31^c.d^	8.4±4.1^d^
**Visfatin (ng/mL)**	7.3±2.8	7.5±3.4	6.6±2.4	7.8±2.3	7.6±2.2
**Fat mass (Kg)**	9.2±2.5^e^	15.8±4.1^d^	23.8±3.9^c^	37.9±6.0^b^	56.7±11.8^a^
**Fat-free mass (Kg)**	39.3±3.6^e^	43.7±3.8^d^	46.9±3.6^c^	53.9±5.2^b^	64.3±8.4^a^
**VFR**	1.06±0.24^d^	2.09±1.2^d^	4.7±0.86^c^	11.3±3.8^b^	16.8±5.9^a^

The results are given as the mean ± SD. Different letters indicate significant differences between the different groups (p<0.05).MO: morbid obesity; BMI: body mass index; HDL-c: HDL-Cholesterol; HOMA-IR: homeostasis model assessment of insulin resistance index; VFR: visceral fat rating.

### Smell and taste measures

**Fig 1:**
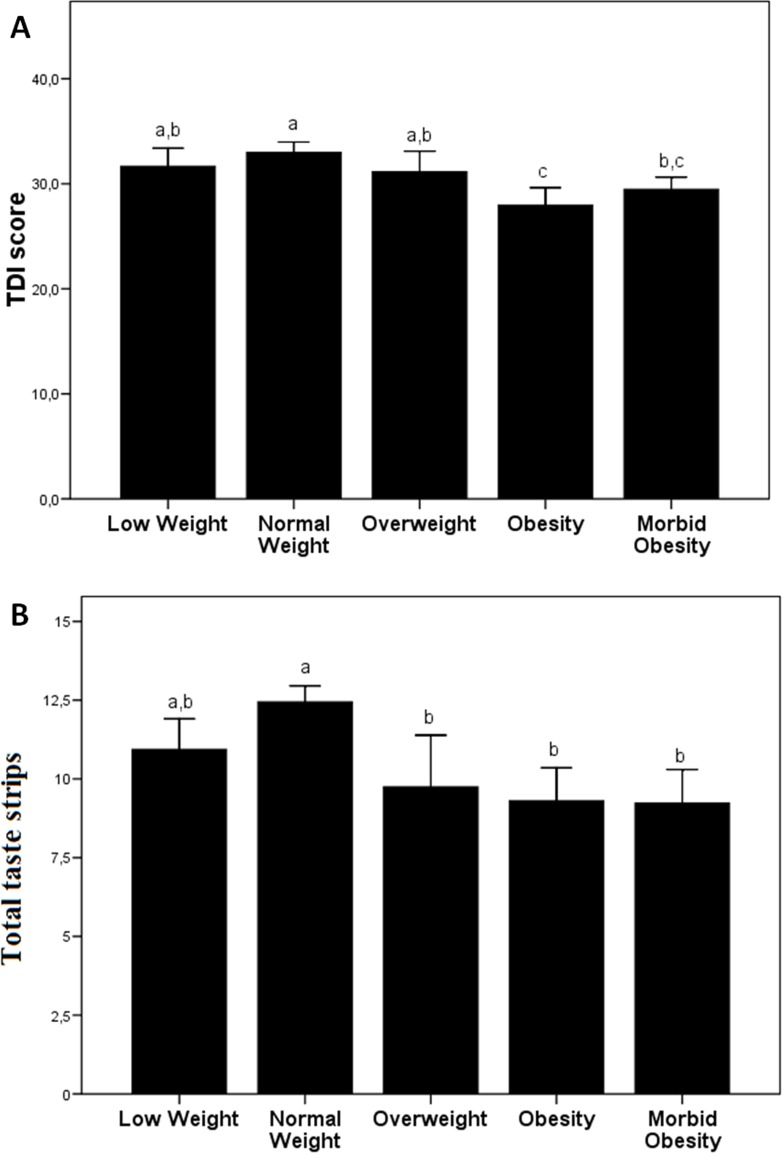
Olfactory and taste function measures of the different groups of subjects included in the study. (A) TDI-score and (B) total taste strips. Different letters indicate significant differences between the different groups (p<0.05). The results are given as the mean ± SEM.

Table 2 summarizes the olfactory and taste function measurements of the different groups of subjects included in the study. We show that the main significant differences in odor threshold, differentiation and identification are found between normal weight and obese subjects, with lower values in the latter group. With the TDI-score, we also found a lower score in the obese and morbidly obese subjects (Fig 1A). In relation to the taste function measurements, we found that normal-weight subjects show a higher score of taste functions, with a tendency to decrease in the groups with greater or lesser BMI (Fig 1B).

**Table 2:** Olfactory and gustatory variables in patients included in the study according to the diagnosis.

VARIABLES	LOW WEIGHT	NORMAL WEIGHT	OVERWEIGHT	OBESITY	MO
**OLFACTORY FUNCTIONS**					
**Sniffin sticks: Odor threshold**	6.0±1.4^a,b^	7.03±2.4^a^	5.5±1.1^b^	5.4±1.6^b^	5.6±1.9^b^
**Sniffin sticks: Odor discrimination**	13.0±1.4^a^	12.9±1.8^a^	12.8±1.9^a^	11.5±1. 9^b^	11.9±2.3^a,b^
**Sniffin sticks: Odor identification**	12.6±1.7^a^	13.0±1.8^a^	12.8±1.6^a^	11.1±2.2^b^	11.9±2.0^a,b^
**GUSTATORY FUNCTIONS**					
**Taste strips sweet**	3.6±0.72^a,b^	3.7±0.63^a^	3.2±0.45^a,b^	3.3±0.77^a,b^	3.0±1.1^b^
**Taste strips acid**	2.0±0.99^b^	2.6±0.93^a^	1.8±0.71^b^	1.9±1.1^b^	1.6±0.93^b^
**Taste strips salty**	2.9±0.99^a^	2.9±0.91^a^	2.3±1.2^a,b^	1.7±1.1^b^	2.4±1.3^a^
**Taste strips bitter**	2.4±1.2^b^	3.1±0.93^a^	2.3±1.6^b^	2.3±1.1^b^	2.2±1.3^b^

The results are given as the mean ± SD. Different letters indicate significant differences between the different groups (p<0.05). MO: morbid obesity. Sniffin' Sticks odor threshold: score range from 0 to 16; Sniffin' Sticks odor discrimination: score range from 0 to 16; Sniffin' Sticks odor identification: score range from 0 to 16. The higher score the better odor performance in each of the subtest. Each correct answer was granted as 1 point (maximum 4 points for each taste score and 16 points for the whole test score). Taste strips sweet: score range from 0 to4; Taste strips acid: score range from 0 to 4; Taste strips salty: score range from 0 to 4; Taste strips bitter: score range from 0 to 4. The higher score the better gustatory function in each of the subtest.

### Relation between smell, taste and anthropometric and biochemical characteristics

**Fig 2:**
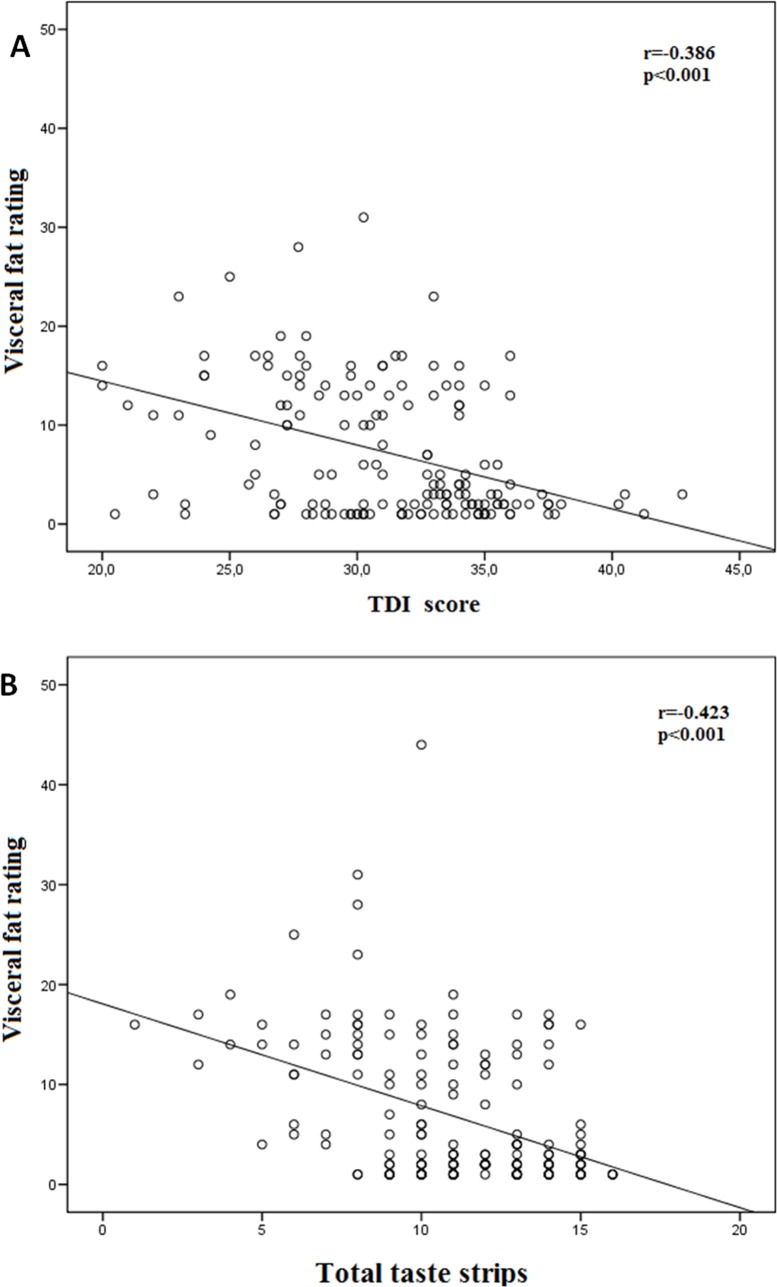
Correlations between visceral fat rating and TDI-score (A) and total taste strips (B).

With regard to the olfactory function, most of the functions measured (threshold, discrimination, identification) and the TDI-score correlated negatively with age, BMI, leptin, fat mass, fat-free mass and VFR (Fig 2A) (Table 3). The TDI-score correlated negatively with visfatin, glucose, cholesterol and triglycerides (Table 3). The odor stimuli threshold correlated negatively with visfatin (Table 3). The odor stimuli discrimination correlated negatively with visfatin and glucose (Table 3). The odor stimuli identification correlated negatively with glucose, cholesterol and triglycerides (Table 3). No other significant correlations were found. In a multiple linear regression model, the only parameter which contributed significantly to explain the TDI-score was the VFR (Table 4).

**Table 3:** Significant Pearson correlations (*p*) between the smell assessments and different biochemical and anthropometrics variables.

VARIABLES	ODOR THRESHOLD	ODOR DISCRIMINATIÓN	ODOR IDENTIFICATION	TDI_SCORE
**AGE**	r = -0.162, p = 0.036	r = -0.221, p = 0.004	r = -0.252, p = 0.001	r = -0.301, p < 0.001
**BMI**	r = -0.211, p = 0.006	r = -0.219, p = 0.005	r = -0.291, p <0.001	r = -0.341, p < 0.001
**LEPTIN**	r = -0.163, p = 0.035	r = -0.246, p = 0.001	r = -0.223, p = 0.004	r = -0.300, p < 0.001
**VISFATIN**	r = -0.181, p = 0.019	r = -0.187, p = 0.015	Ns	r = -0.237, p = 0.002
**GLUCOSE**	Ns	r = -0.175, p = 0.024	r = -0.177, p = 0.023	r = -0.202, p = 0.009
**CHOLESTEROL**	Ns	Ns	r = -0.234, p = 0.003	r = -0.165, p = 0.034
**TRIGLYCERIDES**	Ns	Ns	r = -0.155, p = 0.046	r = -0.194, p = 0.012
**FAT MASS**	r = -0.214, p = 0.006	r = -0.236, p = 0.002	r = -0.329, p <0.001	r = -0.367, p < 0.001
**FAT-FREE MASS**	r = -0.208, p = 0.007	r = -0.157, p = 0.044	r = -0.334, p <0.001	r = -0.329, p < 0.001
**VFR**	r = -0.210, p = 0.007	r = -0.240, p = 0.002	r = -0.363, p <0.001	r = -0.386, p < 0.001

TDI: Threshold, Discrimination and Identification; VFR: visceral fat rating. r = Pearson correlations; p = significance.

**Table 4:** Multiple linear regression model having as dependent variable the TDI_score.

VARIABLES		STANDARDIZED COEFFICIENTS	SIGNIFICANCE
	R^2^	Beta	P
**VFR**	0.149	-0.386	<0.001

Independent variables: age, BMI, leptin, fat mass, fat free mass, VFR and smoking. VFR: visceral fat rating.

Regarding taste functions, most of the functions measured and the total taste strips correlated negatively with age, BMI, leptin, fat mass, fat-free mass and VFR (Fig 2B), and positively with HDL-cholesterol (Table 5). The sweet taste strips correlated negatively with triglycerides, insulin and HOMA-IR (Table 5). The sour taste strips correlated negatively with cholesterol, triglycerides, insulin and HOMA-IR (Table 5). The salty taste strips correlated negatively with glucose, insulin and HOMA-IR (Table 5). The bitter taste strips correlated negatively with glucose and positively with glucose (Table 5). All of the taste strips correlated negatively with glucose, triglycerides, insulin and HOMA-IR and positively with adiponectin (Table 5). No other significant correlations were found. In a multiple linear regression model, the only parameters which contributed significantly to explain the total taste strips were VFR and age (Table 6).

**Table 5:** Pearson correlations (*p*) between the taste assessments and different biochemical and anthropometrics variables.

VARIABLES	T.S.: SWEET	T.S.: SOUR	T.S.: SALT	T.S.: BITTER	TOTAL TASTE STRIPS
**AGE**	r = -0.194, p = 0.011	r = -0.244, p = 0.001	r = -0.441, p < 0.001	r = -0.239, p = 0.002	r = -0.408, p < 0.001
**BMI**	r = -0.301, p < 0.001	r = -0.388, p < 0.001	r = -0.237, p = 0.002	r = -0.239,p = 0.002	r = -0.407, p < 0.001
**LEPTIN**	r = -0.261, p = 0.001	r = -0.264, p < 0.001	r = -0.264, p < 0.001	r = -0.172,p = 0.025	r = -0.338, p < 0.001
**ADIPONECTIN**	Ns	Ns	Ns	r = 0.182,p = 0.017	r = 0.180, p = 0.019
**GLUCOSE**	.Ns	Ns	r = -0.258, p = 0.001	r = -0.160,p = 0.039	r = -0.224, p = 0.004
**CHOLESTEROL**	Ns	r = -0.192, p = 0.013	Ns	Ns	Ns
**TRIGLYCERIDES**	r = -0.166, p = 0.032	r = -0.181, p = 0.019	Ns	Ns	r = -0.219, p = 0.004
**HDL-C**	r = 0.262, p = 0.001	r = 0.213, p = 0.006	r = 0.172, p = 0.026	r = 0.206, p = 0.008	r = 0.298, p < 0.001
**INSULIN**	r = -0.173, p = 0.026	r = -0.217, p = 0.005	r = -0.164, p = 0.035	Ns	r = -0.233, p = 0.003
**HOMA-IR**	r = -0.154, p = 0.048	r = -0.185, p = 0.018	r = -0.182, p = 0.019	Ns	r = -0.218, p = 0.005
**FAT MASS**	r = -0.273, p < 0.001	r = -0.369, p < 0.001	r = -0.226, p = 0.003	r = -0.197, p = 0.010	r = -0.371, p < 0.001
**FAT-FREE MASS**	r = -0.219, p = 0.004	r = -0.304, p < 0.001	r = -0.208, p = 0.007	r = -0.175, p = 0.023	r = -0.319, p < 0.001
**VFR**	r = -0.271, p < 0.001	r = -0.421, p < 0.001	r = -0.266, p = 0.001	r = -0.242, p = 0.002	r = -0.423, p < 0.001

T.S.: taste strips; Total taste strips: point total taste; BMI: body mass index, HDL-c: high density lipoprotein cholesterol; HOMA-IR: homeostasis model assessment of insulin resistance index, VFR: visceral fat rating. r = pearson correlations; p = significance.

**Table 6:** Multiple linear regression model having as dependent variable the total taste strips.

VARIABLES		STANDARDIZED COEFFICIENTS	SIGNIFICANCE
	R^2^	Beta	P
**VFR**	0.179	0.267	0.006
**AGE**	0.207	0.230	0.017

Independent variables: age, BMI, leptin, fat mass, fat free mass, VFR and smoking. VFR: visceral fat rating.

## Discussion

In this research we demonstrate for the first time that visceral fat is related with taste and smell perception in a population of women with different body weights.

Smell plays an important role in the perception of food. Poor odor perception in humans can lead to changes in food consumption, diminished food appreciation and poor nutritional status [36]. We observed that there is a clear decrease of olfactory sensitivity in obese subjects. This result is according to other studies conducted in obese subjects, in both adults and children, in which there is a significant correlation between elevated BMI and the presence of olfactory dysfunction [12,13,37]. Another study shows a marked loss of olfactory sensory function in mice with an induced long-term, diet induced obesity after exposure to a fatty diet [38].

In this study we have found a negative relation between the olfactory function and those variables related to age, BMI, such as leptin, fat mass, fat-free mass VFR. However, when these variables were corrected in a multiple regression model, the amount of visceral fat was the only variable associated with the decrease in olfactory function. This relation can explain the difference with previous studies, in which body composition was not analyzed [12,13,37]. Likewise, subjects with the same BMI have important differences in visceral fat and this aspect could be a cause of differences in research projects that do not analyze this aspect.

It is known that the accumulation of fat in the abdominal area, particularly in the visceral fat compartment, is associated with an increased risk of displaying complications such as insulin resistance, diabetes, dyslipidemias and atherosclerosis [39]. Palouzier-Paulignan et al. [40], in an excellent review of the effects of chronic and acute changes in metabolic signals on olfaction in both humans and animal models, showed that type II diabetes mellitus decrease odor-identification ability and increase detection threshold in humans. On the other hand, it is known that visceral fat acts as a large endocrine gland, excreting cytokines and adipokines which lead to insulin resistance and pro-inflammatory state [41]. The hypothesis that some of these adipokines could have a relation with the alteration in olfactory functions is possible. It has been shown that adipokines may alter or predict the perception and pleasantness of specific odors [24]. In our study we have found a negative association between olfactory function and leptin. This is also according to a study conducted in lean and obese Zucker rats, in which deficits in leptin signaling are associated with an enhanced sensitivity to food cues and that these responses are correlated with food intake and body weight [42]. It has been postulated that there is a leptin resistance in obese subjects. Thus, leptin resistance could increase overeating in part by modulating a person’s sensitivity to food cues [42]. However, not all animal models of obesity result in the same olfactory changes [43]. This suggests that other molecules produced by adipose tissue could be involved in the decrease of olfactory functions.

Regarding taste function, we have found that total taste strips decrease gustatory ability in obesity. According to our results, Pasquet et al. [44] observed that massively obese adolescents have lower thresholds for taste recognition than normal-weight controls. Obrebowski et al. [13] found that children and adolescents with simple obesity have lowered electrogustometric thresholds. Other studies show that obese individuals have a tendency to higher taste thresholds than lean subjects [45], although others show no differences in taste testing between obese and lean subjects [46]. These discrepancies are shown mainly with the sweet taste thresholds. In our study, sweet taste strips show the highest values within each group of subjects, and a negative association has been found between sweet taste and BMI. These results match other research that show how elevated preferences for sweet tastes have been linked with obesity and weight gain in humans [14,15,47]. However, other investigators have found either inverse or no relationship between sweet preference and obesity [4,16,48].

Although the association between BMI and taste has been investigated, we show for the first time that there is a negative relation between total taste strips and BMI-related variables, such as fat mass, fat-free mass, VFR and leptin. Again, only VFR and age are associated with all of the total taste strips in a multiple regression model. This is in agreement with previous studies showing that the gustatory ability decreased with increasing age and was higher in females than in males [36,49]. However, another study found that taste thresholds have been shown to increase with age [50].

Also, an inverse relation has been found between leptin and all of the taste strips (sweet, sour, salty and bitter). Different studies have shown that leptin may act as a modulator of sweet taste responses in mammals, having a role in maintaining energy homeostasis [19,20,23]. The decrease of serum leptin was significantly associated with the decrease in the sweet taste threshold during weight loss in healthy and obese females [21]. It has been suggested that weight loss can lead to an improvement in sweet taste, which may be in part accounted for by the decrease in leptin in obese females [21].

## Limitations

Although the current study addresses a number of methodological gaps in the literature by controlling important potential confounders, there are limitations to consider. First, the current study was conducted solely with female participants and with different sample sizes for groups. Thus, it is unclear whether these findings could be generalized to men. Future studies should also assess male participants and their smell-taste capacity and whether they also showed similar smell dysfunctions and the interaction patterns found in our study. Second, the hormonal profile considered in the study is limited and may expand in future studies to other new hormonal compounds and targets that might be associated. Third, we have not controlled recent food consumption, which may affect sensitivity (particularly olfactory) as shown in previous work of Stafford et al. [51]. Fourth, the use of experimental taste and olfactory stimuli are not reflective of the true perception of a real meal. Fifth and final, we could not perform the oral glucose tolerance test in these patients to define diabetic state, and had not HbA1c levels. We only had the fasting glucose and medication to classify these patients as diabetics. Finally, the visceral fat rating is calculated internally by Tanita Multi Frequency Body Composition Analyzer and visceral fat is not directly measured. However, this non-invasive technique is been extensively used for different research groups as a surrogate for indirect visceral fat quantification [32,33]. Despite the limitations mentioned above, this study has several important strengths, including the significant sample size.

## Conclusions

In summary, this is the first article that relates closely visceral fat with olfactory and gustatory capacity. The search of signals in visceral adipose tissue which trigger this decrease is a task that researchers will have to consider in the following years.
