# Correction: An increase in visceral fat is associated with a decrease in the taste and olfactory capacity

**DOI:** 10.1371/journal.pone.0173588

**Published:** 2017-03-03

**Authors:** Jose Carlos Fernandez-Garcia, Juan Alcaide, Concepcion Santiago-Fernandez, MM. Roca-Rodriguez, Zaida Aguera, Rosa Baños, Cristina Botella, Rafael de la Torre, Jose M. Fernandez-Real, Gema Fruhbeck, Javier Gomez-Ambrosi, Susana Jimenez-Murcia, Jose M. Menchon, Felipe F. Casanueva, Fernando Fernandez-Aranda, Francisco J. Tinahones, Lourdes Garrido-Sanchez

The following information is missing from the Funding section: This study was supported by the project Instituto de Salud Carlos III (FIS PI15/01114).
